# A Unique Case of Penile Necrotizing Fasciitis Secondary to Spontaneous Corpus Cavernosal Abscess

**DOI:** 10.1155/2013/576146

**Published:** 2013-10-02

**Authors:** N. J. Dempster, N. U. Maitra, L. McAuley, M. Brown, D. Hendry

**Affiliations:** Urology Department, Gartnavel General Hospital, 1053 Great Western Road, Glasgow G12 0YN, UK

## Abstract

Corpus cavernosal abscess and necrotizing fasciitis occur rarely, and precipitating factors can usually be elicited with careful history and examination. Whilst both conditions share common risk factors such as diabetes mellitus, this is the first reported case of penile necrotizing fasciitis secondary to spontaneous corpus cavernosal abscess in an otherwise healthy patient. A 32-year-old man presented with 4-day history of swollen, painful penis, with ultrasound confirming corpus cavernosal abscess. Biopsies were taken and the cavity aspirated, but, despite intravenous antibiotics, he developed penile necrotizing fasciitis necessitating open cavernostomy and debridement. The overlying skin defect healed by secondary intention, but the patient experienced persistent postoperative erectile dysfunction, so he was referred for penile prosthesis insertion.

## 1. Introduction

Spontaneous abscesses of the corpus cavernosum are rare, with few previously described idiopathic cases.  The most frequently identified risk factors include relative immunosuppression (e.g., diabetes mellitus) or preceding local or distant infection [1–3]. Penile instrumentation, injection, and trauma have also been described as precipitating factors [4–7]. In this paper we report a case of spontaneous primary corpus cavernosal abscess with subsequent development of penile necrotizing fasciitis. Whilst both conditions share common risk factors [8], to the best of our knowledge this clinical course has not been described in an otherwise healthy patient.

## 2. Case Presentation

A thirty-two-year-old man presented with a four-day history of a swollen and painful penis and left testicle with associated rigors, lower back pain, nausea, and vomiting. There was no history of lower urinary tract symptoms, haematuria, penile discharge, trauma, or unprotected sexual intercourse. His past medical history included mild asthma and hay fever, but he took no regular medications. Abdominal examination was unremarkable, and his genitalia were very swollen and tender on palpation. The patient was pyrexial (38.4°C) and tachycardic (rate 120 b.p.m.) on admission, but blood pressure was stable. Urine dipstick was positive for blood and ketones only. White cell count was 16.24 × 10^9^/L, C-reactive protein was 158 mg/L but urea and electrolytes, liver function tests, glucose and haemoglobin were all within their normal range.

**Figure 1:**
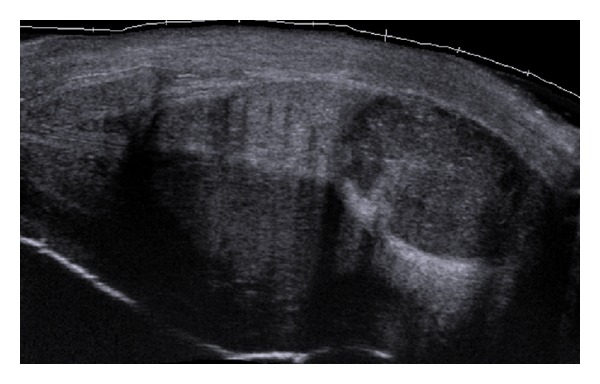
Longitudinal left of midline view of whole penis demonstrating clearly demarcated abscess within the proximal corpus cavernosum.

Empirical intravenous amoxicillin and gentamycin treatment was commenced as per local protocol after discussion with microbiology. Ultrasound showed a 3.5 × 2.5 × 2 cm irregular mass lesion within the base of the left corpus cavernosum with no blood flow, suggestive of either thick pus or a solid lesion (Figure 1). A small volume of blood-stained fluid was aspirated, and tru-cut core biopsies were taken in theatre the following day.

The patient's clinical condition and inflammatory markers initially improved thereafter, but pyrexia and breakdown of the penile skin with purulent discharge were noted 4 days later despite continued intravenous antibiotics. These were changed to tazobactam and piperacillin, and repeat ultrasound scan identified persistent abscess and possible thrombosis of the corpus cavernosum. 

He was taken back to theatre, where an abnormal area on the left ventral aspect of the base of the penis with a line of overlying skin demarcation was excised. Features were typical of necrotizing fasciitis without involvement of the scrotum or perineum. A large abscess cavity was opened, necrotic tissue was debrided to bleeding edges, and the cavity was packed. Cystoscopy was normal.

**Figure 2:**
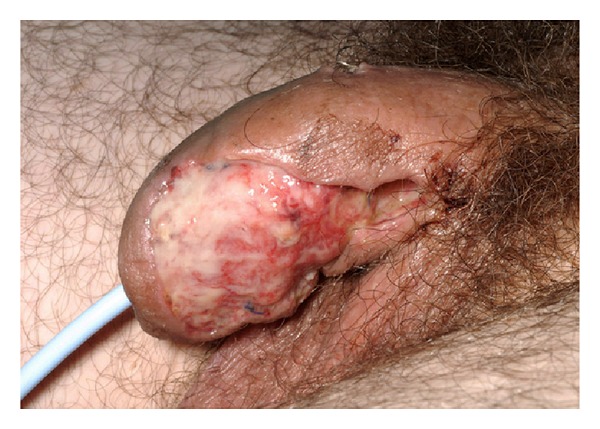
Left lateral view of penis following debridement.

The patient recovered after open cavernostomy and debridement (Figure 2) and was discharged 24 days after admission. Cultures of abscess fluid, urine, and blood were negative. Histopathology of biopsy material showed acute inflammation only. He had a 5 × 4 cm defect at the base of his penis overlying the left corpus cavernosum and was assessed by the plastic surgeons with a view to skin grafting. This proved unnecessary as the wound healed well by secondary intention, but he required penile prosthesis insertion due to postoperative erectile dysfunction.

## 3. Discussion

Abscesses of the corpus cavernosum occur from the neonatal period onwards, which may be unilateral or bilateral and can be associated with priapism at the time of presentation [4, 9]. They are uncommon, and spontaneous cases are of even greater rarity. The most frequently identified risk factors are diabetes mellitus [3], preceding infection [1–3], intracavernosal injection [4, 5], and intravenous drug use involving the external genitalia [6]. Occurrence following insertion of a penile prosthesis [3], “penile fracture” (rupture of the tunica albuginea in the erect penis) [7], and intermittent self catheterisation has also been reported [5].

Initial investigations should include culture of urine, blood, and any discharge or pus prior to antibiotic therapy to maximise the probability of identification of causative organisms, including gonorrhoea, skin, and gastrointestinal commensal organisms. The occasional culture of oral cavity commensals has been associated with distant infection from dental caries [1] and cross-infection from fellatio [2]. Candidal infection has been related to immunocompromise and relative ischaemia due to small vessel disease in a diabetic patient [3]. In our case, there were no positive microbiological cultures, which mirrors around half of those previously reported.

Ultrasound is the most frequently used imaging modality for diagnostic confirmation [2, 4, 5], although urethrogram (voiding or retrograde) [1], computed tomography [10], cavernosography [10], magnetic resonance imaging, and indium-labeled leukocyte scans [10] have also been utilised.

Once the diagnosis has been confirmed, treatment consists of antibiotic therapy, which is usually combined with open cavernotomy to debride necrotic tissue and drain the abscess [1, 4]. When present, any foreign body (e.g., prosthesis) is removed [3]. Suprapubic catheterisation at the time of cavernotomy is also occasionally performed [1]. Percutaneous drainage has been described, but as our case highlights, either repeated aspiration or subsequent cavernotomy and more extensive debridement may be required. Erectile dysfunction and penile curvature secondary to fibrosis rates may, however, be lower with percutaneous management [5]. 

Erectile dysfunction and penile curvature are the most frequently reported sequelae of corpus cavernosal abscess [1, 10]. Extensive debridement of cavernosal tissue, as with our case, commonly precipitates erectile dysfunction and may be treated with a penile prosthesis. Abscess recurrence may occur several months after primary treatment [3], and scrotal abscess [1] and urethral sinuses have also been reported. To the best of our knowledge there has been only one reported case of corpus cavernosal abscess with subsequent development of penile necrotizing fasciitis. This occurred three weeks after false penile fracture, and early haematoma evacuation is recommended in such cases. The patient was therefore at increased risk of local infection due to his delayed presentation and penile trauma [7]. In contrast, in our case no risk or precipitating factors were present.

In conclusion, our case illustrates that the serious complication of necrotizing fasciitis may occur after corpus cavernosal abscess in an otherwise healthy patient. Early surgical intervention is therefore recommended as definitive treatment in all cases of corpus cavernosal abscess to prevent its development.
